# Ascorbate Attenuates Oxidative Stress and Increased Blood Pressure Induced by 2-(4-Hydroxyphenyl) Amino-1,4-naphthoquinone in Rats

**DOI:** 10.1155/2018/8989676

**Published:** 2018-07-26

**Authors:** Javier Palacios, José Miguel Fonseca, Fernando Ayavire, Felipe Salas, Mirko Ortiz, Juan Marcelo Sandoval, Julio Benites, Chukwuemeka R. Nwokocha, Ewaldo Zavala, Adrián Paredes, Iván Barría, José Luis Vega, Fredi Cifuentes

**Affiliations:** ^1^Departamento de Ciencias Químicas y Farmacéuticas, Facultad Ciencias de la Salud, Universidad Arturo Prat, Iquique, Chile; ^2^Department of Basic Medical Sciences, Physiology Section, Faculty of Medical Sciences, The University of the West Indies, Mona, Kingston 7, Jamaica; ^3^Facultad de Farmacia y Bioquímica, Universidad Nacional de Trujillo, Trujillo, Peru; ^4^Laboratorio de Química Biológica, Instituto Antofagasta (IA), Universidad de Antofagasta, Antofagasta, Chile; ^5^Laboratorio de Fisiología Experimental (EPhyL), Instituto Antofagasta (IA), Universidad de Antofagasta, Antofagasta, Chile

## Abstract

Quinone derivatives like 2-(4-hydroxyphenyl) amino-1,4-naphthoquinone (Q7) are used as antitumor agents usually associated with adverse effects on the cardiovascular system. The objective of this study was to evaluate the cardioprotective effect of ascorbate on Q7-induced cardiovascular response in Wistar rats. In this study, blood pressure, vascular reactivity, and intracellular calcium fluxes were evaluated in cardiomyocytes and the rat aorta. We also measured oxidative stress through lipid peroxidation (TBARS), superoxide dismutase- (SOD-) like activity, and H_2_O_2_ generation. Oral treatment of rats with ascorbate (500 mg/kg) for 20 days significantly (*p* < 0.05) reduced the Q7-induced increase (10 mg/kg) in blood pressure and heart rate. The preincubation with ascorbate (2 mM) significantly (*p* < 0.05) attenuated the irregular beating of the atrium induced by Q7 (10^−5^ M). In addition, ascorbate induced endothelial vasodilation in the presence of Q7 in the intact aortic rings of a rat and reduced the cytosolic calcium levels in vascular smooth muscle cells. Ascorbate also reduced the Q7-induced oxidative stress *in vivo*. Ascorbate also attenuated Q7-induced SOD-like activity and increased TBARS levels. These results suggest a cardioprotective effect *in vivo* of ascorbate in animals treated orally with a naphthoquinone derivative by a mechanism involving oxidative stress.

## 1. Introduction

Naphthoquinone derivatives are widely distributed molecules in nature. Numerous antitumor therapeutic drugs are quinone-bearing molecules; these include anthracyclines, the 1,4-naphthoquinone pharmacophore group, and several synthetic compounds [1–4]. The therapeutic spectrum of action of quinone derivatives is very wide: leukemia, breast and lung cancer, lymphomas, and others [5].

Treatment of cancer with anthracycline derivatives has been very successful. However, these treatments generate increased cardiotoxic effects such as hypertension, heart failure, vascular complications, and cardiac arrhythmia [6]. Oxidative stress, DNA damage, senescence, and cell death are mechanisms causing anthracycline toxicity [7]. Cytotoxic and cardiotoxic effects of naphthoquinone derivatives involve the generation of reactive oxygen species (ROS) by a redox-cycling reaction [8–11]. Redox-cycling reaction occurs through quinone reduction by 1 or 2 electrons from NADPH cytochrome P450 reductase, leading to a semiquinone-free radical that is reoxidized to the quinone in the presence of molecular oxygen, while oxygen is reduced to superoxide anion [12].

To reduce the cardiotoxic effects of anthracycline derivatives, researchers have evaluated its coadministration with molecules displaying antioxidant capacity. L-Carnitine supplementation was shown to reduce antioxidant defense with doxorubicin administration [13, 14]. In contrast, ascorbate plays a cardioprotective role in doxorubicin-induced cardiomyopathy by decreasing oxidative and/or nitrosative stress [15]. Phytochemical metabolites prevent oxidative stress by decreasing ROS generation, free radical scavenging activity, or improving the antioxidant effect of cells [16].

Ascorbate increases nitric oxide (NO) bioavailability in vascular endothelial tissue from dysfunctional patients. The protective effect of ascorbate on the vascular endothelium has been linked to the enhanced bioavailability of the tetrahydrobiopterin (BH_4_) or the endothelial nitric oxide synthase (eNOS) activity [17]. The key role of an antioxidant agent relies on its ability to donate one or two electrons [18].

A previous study from our group showed that arylamino-naphthoquinone derivatives like Q7 (2-(4-hydroxyphenyl) amino-1,4-naphthoquinone) increased the formation of ROS and impaired the endothelial vasodilation in the rat aorta [19]. The objective of this investigation was to evaluate possible cardioprotective effects of ascorbate on the cardiotoxic response induced through chronic treatment with a naphthoquinone derivative Q7.

## 2. Materials and Methods

### 2.1. Drugs

The following drugs were used in this study: 2-(4-hydroxyphenyl) amino-1,4-naphthoquinone (Q7); acetylcholine (Sigma-Aldrich, USA); ascorbate (Asc) (Winkler, Santiago); phenylephrine (Sigma-Aldrich, USA); butylated hydroxytoluene (Merck, Darmstadt, Germany); pyrogallol (Sigma-Aldrich, USA); tetramethoxypropane (Sigma-Aldrich, USA); thiobarbituric acid (Merck, Darmstadt, Germany); and Tris-cacodylic acid (Sigma-Aldrich, USA). Drugs were dissolved in distilled deionized water. Acetylcholine solution in Krebs-Ringer bicarbonate (KRB) buffer was freshly prepared before each experiment.

### 2.2. Animals

Male and female Wistar rats (4 weeks of age, 150–170 g) from the Height Institute of Arturo Prat University of Iquique were used for this study. The animals were housed in light-cycled (8:00 to 20:00 hours) and temperature-controlled rooms. In addition, the rats were provided ad libitum access to drinking water and standard rat chow (Champion, Santiago). Since the female rats were sexually immature [20], no stages of the estrus cycle were observed by vaginal smear. In this study, 25 rats were randomly assigned into five groups of 5 animals each.

#### 2.2.1. *In Vivo* Experiments

These include noninvasive blood pressure and ECG measurements. The oral treatment of animals consisted in a daily administration of a mixture of Q7 and/or ascorbate plus peanut butter for 20 days.

- Group 1 (*n* = 5; control) consists of rats treated with vehicle (peanut butter).
- Group 2 (*n* = 5; Q7) consists of rats treated with Q7 (10 mg/kg).
- Group 3 (*n* = 5; Q7 + Asc) consists of rats treated with Q7 (10 mg/kg) plus ascorbate (500 mg/kg).
- Group 4 (*n* = 5; Asc) consists of rats treated with ascorbate (500 mg/kg).

#### 2.2.2. *In Vitro* Experiments

This includes contractibility measurements in the isolated rat right atrium and thoracic aorta (Group 5, *n* = 5). Cytosolic calcium levels and H_2_O_2_ production were measured in rat cardiomyocytes and A7r5 cells. The tissues or cells were preincubated with Q7 (10^−5^ M) and/or ascorbate (0.125, 0.25, and 2 mM).

For groups 2, 3, and 4, the doses of Q7 and ascorbate were selected according to previous experiments using ECG of the normotensive rats in our laboratory and antitumor activity in mice was also observed [21]. For *in vitro* studies, the concentration of Q7 and ascorbate was selected according to our vascular reactivity experiments in the rat aorta [19].

The experiments of this study were conducted following the ARRIVE guidelines and Guide for the Care and Use of Laboratory Animals published by the National Institute of Health of the United States (NIH, publication revised in 2013) and the Ethics Committee of Arturo Prat University (CEC-17).

### 2.3. Blood Pressure and ECG Recording

The blood pressure and ECG measurements were simultaneously carried out on the rats, as previously described in our laboratory [22]. SBP was measured using the tail-cuff method (BIOPAC Systems) and AcqKnowledge 3.9.1 computer software program. The method of Erken et al. [23] was followed for measuring blood pressures. Animals were acclimatized for 20 min prior to the beginning of the experiment at room temperature (30–33°C). Then the pressure sensor cuffs and plethysmograph were placed on the tail of the animal. An average of 4 reading cycles of blood pressure measurements was made with the conscious animal per day.

For ECG recordings, the animals were anesthetized with ketamine (42 mg/kg, i.p.) and xylazine (5 mg/kg, i.p.). The needle electrodes were placed subcutaneously in bipolar configuration (DII). Measurements were done using the electrocardiographic amplifier (ECG100C BIOPAC) equipment and AcqKnowledge 3.9.1 computer software program.

### 2.4. Frequency of the Isolated Right Atrium

The isolated atrium experiment was carried out on rats as previously described in our laboratory [22]. The heart was isolated and placed in a cold (4°C) Krebs-Ringer bicarbonate (KRB) physiological buffered solution containing (×10^−3^ M) 4.2 KCl, 1.19 KH_2_PO_4_, 120 NaCl, 25 Na_2_HCO_3_, 1.2 MgSO_4_, 1.3 CaCl_2_, and 5 D-glucose (pH 7.4, 37°C, 95% O_2_, and 5% CO_2_). The isolated right atrium of the rat was carefully fixed with a silk thread into a moveable and static lever; the upper one was attached to an isometric transducer (Radnoti, California). PowerLab 8/35 (USA) was used for continuous recording of vascular tension (LabChart 8 program, ADInstruments). The passive tension of the atrium was 0.5 g.

### 2.5. Isolation of Neonatal Cardiac Myocytes

Neonatal rat cardiac myocytes were prepared as previously described [24]. Animals were decapitated and hearts were removed. Then atria were excised and the ventricles were minced in ice-cold PBS without Ca^2+^/Mg^2+^. Ventricular tissue was transferred to a new tube and subjected to overnight digestion (4°C, constant shaking) with 0.05% trypsin-EDTA solution (Gibco, NY, USA). A pelleted fraction was then subjected to digestion using collagenase type II (0.75 mg·mL^−1^, 20 min) (Gibco, NY, USA). The collected enzyme solution was centrifuged, the supernatant was discarded, and the cardiac myocyte fraction was resuspended in a culture medium (DMEM high glucose/M199, 4 : 1) and preplated into a 60 mm dish for 1–3 hours. The preplating step removed fibroblasts and endothelial cells. Nonadherent cells were then collected, quantified, and plated in 2% gelatin-coated 35 mm glass bottom microdish (Ibidi GmbH, Munich, Germany). 3 *μ*g/mL cytosine beta-D-arabinofuranoside was added to the culture medium for 12 hours to eliminate residual fibroblasts. Cultures were maintained in a humidified incubator at 37°C and 5% CO_2_. Culture medium was changed every day.

### 2.6. Monitoring Cytosolic Ca^2+^ Signal on Isolation of Rat Neonatal Cardiac Myocytes

Cytosolic calcium was determined in rat neonatal cardiomyocytes according to Barría et al. [24]. The cells were washed with Krebs-Ringer bicarbonate buffer (KRB) containing (×10^−3^ M) 4.2 KCl, 1.19 KH_2_PO_4_, 120 NaCl, 25 Na_2_HCO_3_, 1.2 MgSO_4_, 1.3 CaCl_2_, and 5 D-glucose (pH 7.4). Then they were loaded with 10^−5^ M Fluo-3 AM for 25 min at 37°C and then were again washed with KRB and incubated for 15 minutes at 37°C. Cells were studied in duplicate, preincubating for 20 min with Q7 (10^−5^ M) and/or ascorbate (2 mM) and then stimulating with phenylephrine (PE, 10^−6^ M). Fluo-3 fluorescence (506 nm excitation, 526 nm emission) was monitored every 5 seconds using a Leica TCS SP8 confocal microscope (Leica, Canada). Fluorescence intensity was analyzed by the ImageJ software (NIH software), which measured the selected region of interest (ROI). Fluorescence was expressed as arbitrary units, and for each value, the background intensity was subtracted.

### 2.7. Isolation of the Aortic Rings

Vascular reactivity was evaluated in the aortic rings according to Paredes et al. [25]. Rats were sacrificed by cervical dislocation. The thoracic aorta was quickly excised and placed in cold (4°C) physiological Krebs-Ringer bicarbonate buffer (KRB) containing (×10^−3^ M) 4.2 KCl, 1.19 KH_2_PO_4_, 120 NaCl, 25 Na_2_HCO_3_, 1.2 MgSO_4_, 1.3 CaCl_2_, and 5 D-glucose and 1 liter of distilled water (pH 7.4). Rings (3-4 mm) were cut after connective tissue was cleaned out from the aorta, taking care to avoid endothelial damage. The aortic rings were equilibrated for 40 min in KRB at 37°C by constant bubbling with 95% O_2_ and 5% CO_2_.

### 2.8. Vascular Reactivity Experiments

To evaluate the vascular function of the endothelium, the vasodilation in response to 10^−5^ M ACh (muscarinic receptor agonist) in the precontracted aortic rings with 10^−6^ M PE was tested. According to the general use of the rat aorta as a pharmacological tool for *in vitro* analysis, the aortic rings were considered for a functional endothelial response if vasodilation was up to 70–80% [26]. Two aortic rings from the same animal were simultaneously studied in different organ baths, using different vasoactive substances (phenylephrine (PE), KCl, and acetylcholine (ACh)). The aortic rings were mounted on two 25-gauge stainless steel wires; the upper one was attached to an isometric transducer (Radnoti, California, USA). The volume of the organ bath was 10 mL. The transducer was connected to a PowerLab 8/35 (Colorado Springs, CO) for continuous recording of vascular tension using the LabChart Prov 8.1.2 computer program (ADInstruments). After the equilibration period for 40 min, the aortic rings were stabilized by three successive near-maximum contractions with KCl (6 × 10^−2^ M) for 10 min. The passive tension on the aortic rings was 1.0 g, which was determined with 6 × 10^−2^ M KCl [27].

### 2.9. Cytosolic Ca^2+^ Signal on Vascular Smooth Muscle Cell (A7r5)

A7r5 cells were cultured in 35 mm culture dish according to the methodology described by Palacios et al. [19]. The cells were washed with Krebs-Ringer bicarbonate buffer (KRB) containing (×10^−3^ M) 4.2 KCl, 1.19 KH_2_PO_4_, 120 NaCl, 25 Na_2_HCO_3_, 1.2 MgSO_4_, 1.3 CaCl_2_, and 5 D-glucose (pH 7.4). They were preincubated with 10^−5^ M Fluo-3 AM for 25 min at 37°C and then were again washed with KRB. Cells were studied in duplicate, preincubating for 20 min with Q7 (10^−5^ M) and/or ascorbate (2 mM) and then stimulating with phenylephrine (PE, 10^−6^ M). A fluorescence microplate reader equipped for excitation in the range of 506 nm and emission detection at 526 nm was used (Infinite® 200 TECAN (Männedorf, Switzerland).

### 2.10. Determination of Lipid Peroxidation Products in Serum

The thiobarbituric acid reactive substances (TBARS) were measured in serum of rat, according to Cifuentes et al. [27]. Briefly, 100 *μ*L of the serum (10.9 ± 0.6 mg protein/mL) was taken and mixed with 200 *μ*L of trichloroacetic acid 10% (TCA) and butylated hydroxytoluene 4% (BHT); this mixture was centrifuged, and then 140 *μ*L of the supernatant was mixed with thiobarbituric acid (0.67%) and heated to 95°C for 1 hour. After cooling to room temperature, 280 *μ*L of butanol-pyridine (15 : 1) was added and mixed and centrifuged at 3000*g* for 20 minutes; finally, the absorbance of the supernatant was measured at 532 nm in an Infinite 200 TECAN (Männedorf, Switzerland).

### 2.11. Determination of Superoxide Dismutase- (SOD-) Like Activity in Serum

The SOD-like activity in serum was determined according to the methodology by Marklund and Marklund [28]. Briefly, 20 *μ*L of the serum (10.9 ± 0.6 mg protein/mL) was taken and mixed with 130 *μ*L of 50 mM Tris-cacodylic acid buffer (pH 8.2), 20 *μ*L of 1 mM EDTA, and 7 *μ*L of pyrogallol (2 mM in 10 mM HCl). The absorbance was monitored at 420 nm and 37°C for 3 min with an Infinite 200 TECAN (Männedorf, Switzerland) against a reagent blank. The SOD-like activity was calculated as the change in absorbance per minute in the experimental sample versus the control sample. The SOD-like activity was expressed according to the equation % = (*E* − *C*) × 100/*E*, where *E* and *C* are autoxidation rates of pyrogallol in an experimental sample and control sample, respectively. When the activity was 100%, pyrogallol autoxidation was completely inhibited.

### 2.12. Determination of H_2_O_2_ in Vascular Smooth Muscle Cell

Vascular smooth muscle cells (A7r5) were cultured in petri dishes (35 mm). High-glucose and Dulbecco's modified Eagle's medium (DMEM) pyruvate was used as a medium culture. The Amplex® Red Kit (Invitrogen) was used for determination of H_2_O_2_ in culture cell, according to Song et al. [29]. The kit contains 10-acetyl-3,7-dihydroxyphenoxazine (Amplex Red) to detect H_2_O_2_. A fluorescence microplate reader equipped with Infinite 200 TECAN (Männedorf, Switzerland) for excitation in the range of 530–560 nm and emission detection at ~590 nm was used.

### 2.13. Statistical Analysis

GraphPad Prism 5 software was used, 𝑛 represents the number of animals studied, and values were expressed as the mean ± standard error of the mean (SEM). For the statistical analysis of the groups, a one-way or two-way ANOVA was used as appropriate, followed by a Bonferroni post hoc test when necessary. A value *p* of <0.05 was considered statistically significant.

## 3. Results

### 3.1. Ascorbate Reverses the Increased Blood Pressure and Heart Rate Induced by Q7

**Figure 1:**
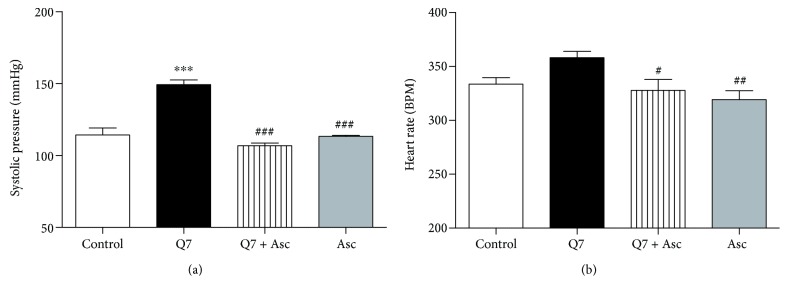
Hypotensive and bradycardic effects of ascorbate on normotensive rats chronically treated with Q7. The results show that oral administration of 500 mg/kg ascorbate (Asc) for 20 days decreased the systolic blood pressure (SBP) (a) and heart rate (HR) (b) in Q7-treated rats (10 mg/kg Q7). Values are mean ± standard error of the mean of 5 experiments in mmHg or BPM. Statistically significant differences: ^∗∗∗^*p* < 0.001 versus control; ^#^*p* < 0.05; ^##^*p* < 0.001; and ^###^*p* < 0.01 versus Q7.

To determine whether the findings of this study may have clinical implications, we measured arterial blood pressure in rats *in vivo*. As shown in Figure 1(a), the chronic treatment with Q7 (10 mg/kg) significantly increased the SBP compared to those of control rats (120 ± 1 mmHg control versus 149 ± 3 mmHg with Q7; *p* < 0.001). In contrast, chronic treatment with ascorbate (Asc) (500 mg/kg) after Q7 exposure significantly reduced the SBP (107 ± 2 mmHg with Q7 + Asc; *p* < 0.001), a similar value to that observed in rats treated with ascorbate alone (113 ± 1 mmHg with Asc; *p* < 0.001).

On the other hand, Figure 1(b) shows that chronic oral administration of ascorbate following Q7 treatment significantly reduced the heart rate (325 ± 6 BPM control versus 243 ± 3 BPM with Q7 + Asc; *p* < 0.05). Q7 treatment caused an increased HR compared with the control rats (364 ± 12 BPM with Q7).

### 3.2. Ascorbate Protects against the Negative Effect of Q7 on the Frequency of the Atrium

**Figure 2:**
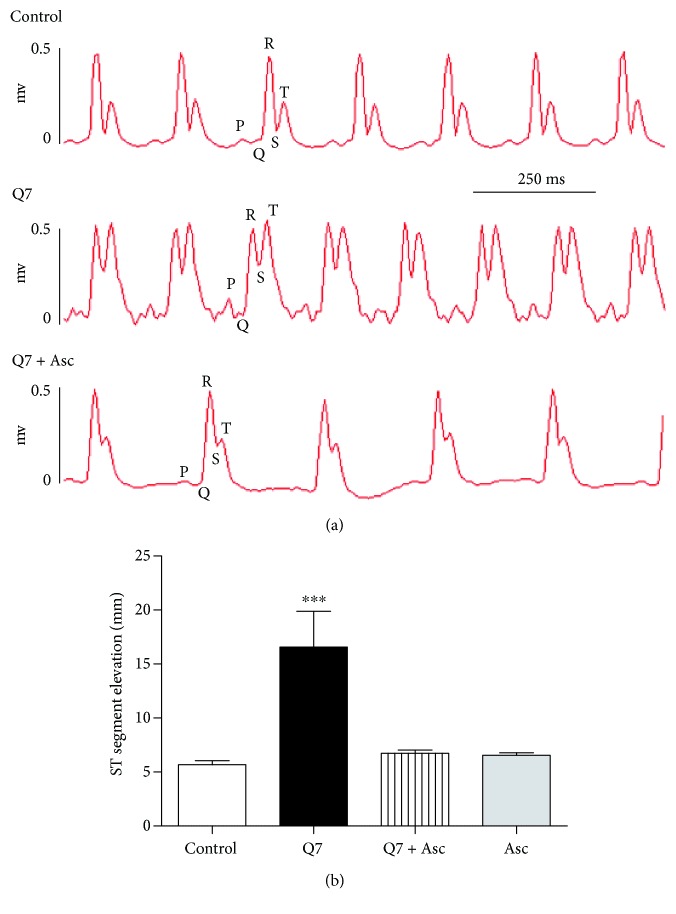
Original trace showing the electrocardiogram (a) and ST segment elevation in rats (b). Oral administration of ascorbate (500 mg/kg Asc) for 20 days decreased the ST segment elevation in Q7-treated rats (10 mg/kg Q7). Values are mean ± standard error of the mean of 5 experiments. Statistically significant differences: ^∗∗∗^*p* < 0.001 versus control.

**Figure 3:**
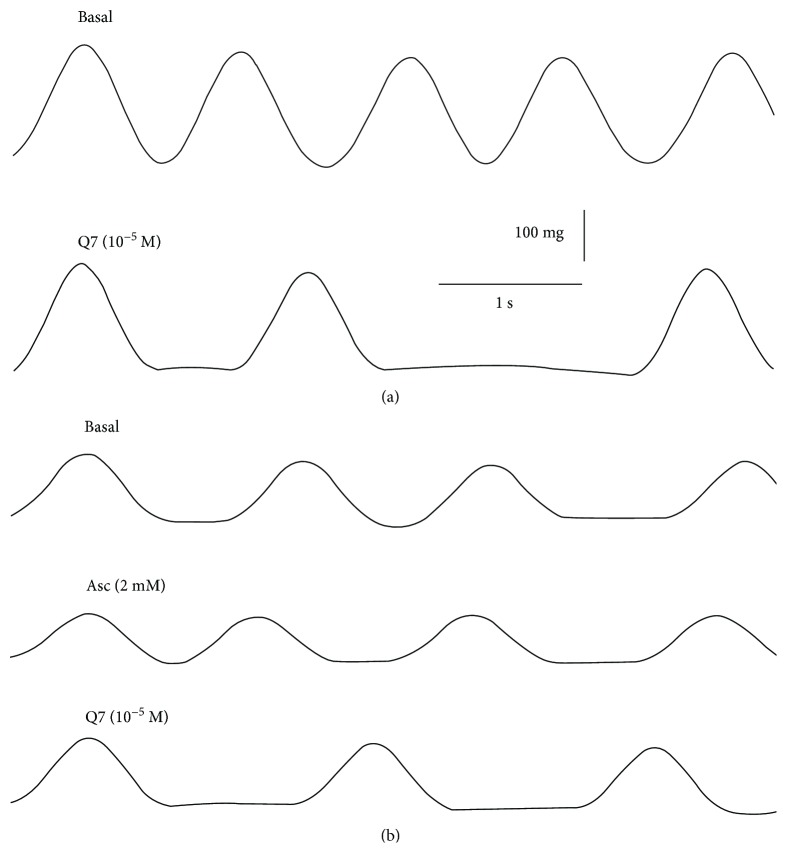
Original trace showing the time course of the frequency of the isolated right atrium. The addition of Q7 to the organ bath caused an irregular beating of the atrium (a), but the preincubation with 2 mM ascorbate before addition of 10^−5^ M Q7 induced regular-frequency beats of the atrium (b). Three independent experiments were performed.

Although no mortality was detected in rats treated with Q7 at the dose used during this study, we found an elevation of ST segment observed in ECG from Q7-treated rats. As seen in Figure 2, oral administration of Q7 (10 mg/kg) significantly caused an elevation of ST segment (5.69 ± 0.36 mm control versus 16.58 ± 3.32 mm Q7 treated; *p* < 0.001). Ascorbate (500 mg/kg) decreased the elevation of ST segment in the presence of Q7 (6.75 ± 0.29 mm). We also evaluated the effect of ascorbate on the beating of the isolated rat atrium. As shown in Figure 3(a), acute treatment with 10^−5^ M Q7 induced an irregular beating of the isolated atrium of the rat, which was prevented when the isolated atria were preincubated with 2 mM ascorbate before addition of 10^−5^ M Q7 (Figure 3(b)).

### 3.3. Ascorbate Effects on the Increased Intracellular Calcium Induced by Q7 in the Cardiomyocytes

**Figure 4:**
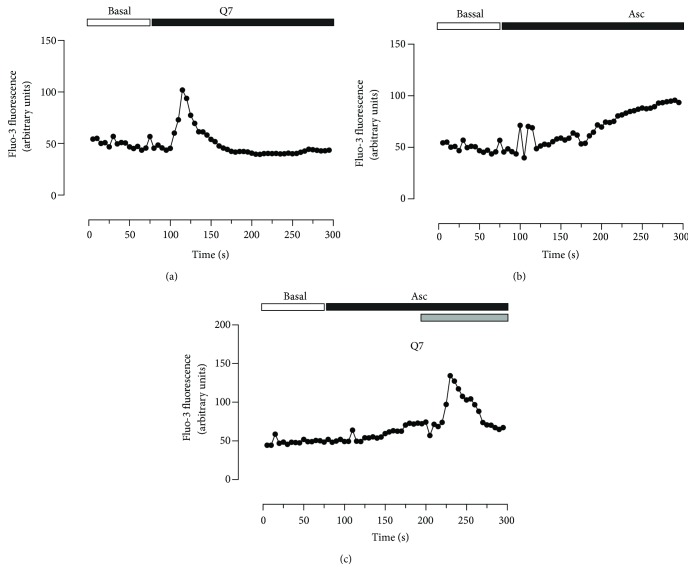
Effects of quinone and ascorbate on intracellular Ca^2+^ levels in rat cardiomyocytes. Quinone Q7 (10^−5^ M) increased intracellular calcium levels in rat cardiomyocytes (a), but ascorbate (Asc) (2 mM) did not prevent the increase of Q7-induced intracellular calcium (c). Effect of ascorbate on intracellular calcium levels in rat cardiomyocytes (b). Three independent experiments were performed.

To investigate if the Q7-mediated effects were dependent on changes in intracellular calcium levels, we measured intracellular calcium levels in isolated rat cardiomyocytes. As shown in Figure 4, the administration of 10^−5^ M Q7 increased the intracellular calcium levels in rat cardiomyocytes, an effect that was not modified in the presence of 2 mM ascorbate. Moreover, an increase in intracellular calcium levels was also observed in cardiomyocytes perfused with 2 mM ascorbate but the kinetics was substantially slower than that observed in response to Q7 (Figure 4(c)).

### 3.4. Ascorbate Improves the ACh-Induced Vasodilation and Decreases the Intracellular Calcium Level in the Presence of Q7 in the Rat Aorta

**Figure 5:**
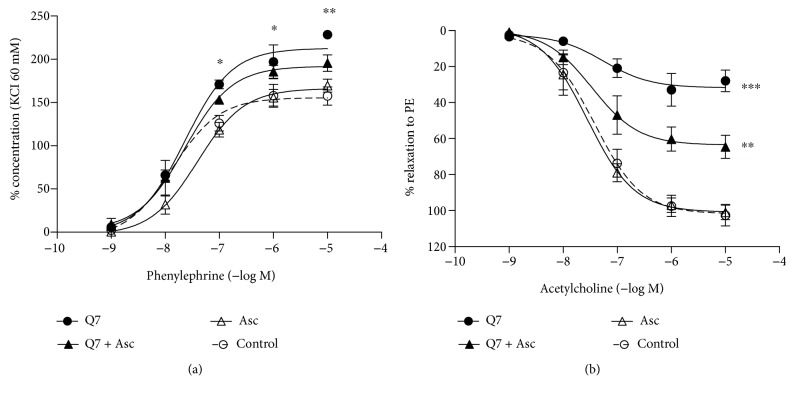
Effect of Q7 and ascorbate on the vascular response in the intact rat aorta. The quinone increased the contractile response to PE and impaired ACh-induced vasodilation. The concentration-response curves to PE (10^−9^ to 10^−5^ M) (a) and ACh (10^−9^ to 10^−5^ M) in the intact aortic rings of rats (b) in the presence or absence (control) of 10^−5^ M Q7 or 2 mM ascorbate (Asc). Arteries were preincubated with Q7 or ascorbate for 30 min. Values are mean ± standard error of the mean of 5 experiments. Statistically significant differences: ^∗^*p* < 0.05, ^∗∗^*p* < 0.01, and ^∗∗∗^*p* < 0.001 versus control.

It is possible that the increase of blood pressure would occur through an increase of the vasoconstriction. Therefore, we analyzed the vascular response in the aortic rings of rats treated with Q7 and/or ascorbate. As shown in Figure 5(a), the vascular contractile response of the intact aortic rings to the alpha-adrenergic receptor agonist PE was significantly increased with Q7 (158 ± 11% control versus 228 ± 3% with Q7; PE 10^−5^ M; *p* < 0.01). Moreover, ascorbate slightly decreased the vascular contractile response to PE in the presence of Q7 (196 ± 10% with Q7 + Asc; PE 10^−5^ M). The aortic rings preincubated with 2 mM ascorbate (170 ± 8%) showed a similar response to PE compared with those of the control experiments.

We also assessed the endothelial ACh-induced vasodilatation in the intact aortic rings. As shown in Figure 5(b), the intact aortic rings in the presence of 2 mM ascorbate showed a complete relaxation in response to different doses of the muscarinic receptor agonist ACh (10^−8^ to 10^−5^ M). Preincubation of intact rings with 10^−5^ M Q7 significantly decreased the ACh-induced vasodilation (102 ± 6% control versus 28 ± 6% with Q7; ACh 10^−5^ M; *p* < 0.001). This negative effect of Q7 on the ACh-induced vasodilation was reverted, in part, by preincubation with ascorbate (65 ± 6% with Q7 + Asc; ACh 10^−5^ M). Nevertheless, sodium nitroprusside (SNP), a NO donor compound, caused a complete vasodilation even in the presence of 10^−5^ M Q7. These data were recently published from our laboratory [19].

In order to gain insight into the potential role of Q7 and ascorbate in the vascular contractile response to PE, determination of intracellular calcium was carried out in vascular smooth muscle cell culture (A7r5). We observed that preincubation of A7r5 cells with Q7 increased the intracellular calcium levels in response to 10^−6^ M PE (Table 1). In agreement with vascular contractile response to PE, preincubation with 2 mM ascorbate attenuated the increase in intracellular calcium levels caused by Q7. Ascorbate per se did not increase the intracellular calcium levels compared to control experiments (Table 1).

### 3.5. Increase of Oxidative Stress in Rats Treated with Q7: TBARS and SOD-Like Activity

Since 1,4-naphthoquinone derivatives produce oxidative stress, we analyzed the oxidative stress in serum samples from Q7-treated rats. TBARS assay and SOD-like activity were used as indicators of oxidative stress. As shown in Table 2, oral administration of Q7 significantly increased the TBARS in serum (26 ± 1 nM control versus 33 ± 2 nM Q7 treated; *p* < 0.05). In the same groups of animals, we confirmed that Q7 significantly decreased the SOD-like activity in serum (159 ± 49% control versus −193 ± 100% Q7 treated; *p* < 0.05). When the activity was ≥100%, pyrogallol autoxidation was completely inhibited, while the negative value means that adding Q7 accelerated it. Since the Q7 + Asc group did not significantly increase the TBARS level (Table 2) or decrease SOD-like activity in serum, suggesting that ascorbate treatment partially reduced the oxidative stress induced by Q7, the Asc-treated group did not show a significant increase in the TBARS level or decrease in SOD-like activity in serum samples compared to control samples.

### 3.6. Ascorbate, but Not Q7, Increases Production of H_2_O_2_ in A7r5 Cells

**Figure 6:**
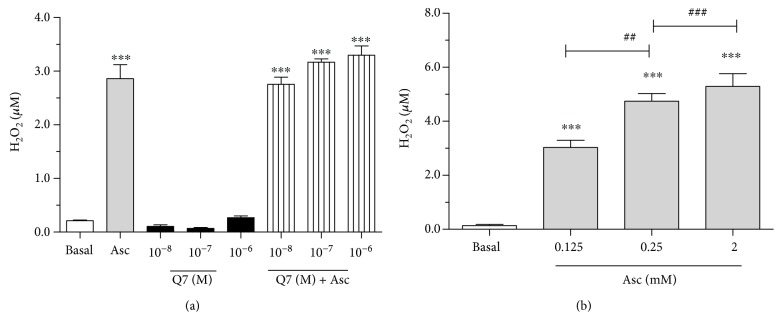
Effect of ascorbate and Q7 on the production of H_2_O_2_ in vascular smooth muscle cells (A7r5). The data shows the H_2_O_2_ generation in the presence of ascorbate and Q7 in A7r5 cells (a) and the effect of increasing doses of ascorbate on H_2_O_2_ generation (b). Ascorbate increased significantly the generation of H_2_O_2_ in a dose-dependent manner in A7r5 cells, while Q7 did not cause any change. Values are mean ± standard error of the mean of 3 experiments. Statistically significant differences: ^∗∗∗^*p* < 0.001 versus basal; ^##^*p* < 0.01, and ^###^*p* < 0.001 versus 0.125 mM Asc.

It has been suggested that Q7 induces ROS (O_2_^−^ and H_2_O_2_) by a redox-cycling mechanism [11]. We measured H_2_O_2_ levels in A7r5 cells in response to Q7. As shown in Figure 6(a), Q7 (10^−8^ to 10^−6^ M) did not increase the generation of H_2_O_2_ compared with basal levels (Figure 6(a)). However, ascorbate alone or in combination with Q7 (10^−8^ to 10^−6^ M) significantly increased (*p* < 0.001) the generation of H_2_O_2_ (0.21 ± 0.01 nM basal versus 2.76 ± 0.13 nM with 10^−8^ Q7; *p* < 0.001).  Figure 6(b) shows that the effect of ascorbate on the generation of H_2_O_2_ was dose dependent (0.125, 0.25, and 2 mM).

## 4. Discussion

The focus of this study was to evaluate whether ascorbate counteracts the adverse cardiovascular effects and oxidative stress induced by a quinone derivative treatment. We found that oral treatment with ascorbate at physiological concentrations reduced the Q7-induced blood pressure in rats. This decrease in blood pressure was as a result of decreased HR and improved endothelial vasodilation in the intact aortic rings of the rat.

The increased Q7-induced SBP was significantly decreased after the treatment with ascorbate. The effect of Q7 is in agreement with other studies, which reported an increased SBP in juglone- (5-hydroxy-1,4-naphthoquinone-) treated mice for 14 days [30]. On the one hand, Q7 could increase the blood pressure by a mechanism dependent on oxidative stress. In fact, Q7 significantly increased lipid peroxidation (TBARS) and decreased the SOD-like activity in serum of rats. In previous studies, we demonstrated increased TBARS levels in rat aorta tissue [19] and in calf thymus DNA treated with Q7 [21]. These findings are in agreement with other reports showing that oral administration of high-dose ascorbate reduced the blood pressure and heart rate in dogs displaying ROS-induced myocardial damage [31].

It is possible that Q7-induced increase of blood pressure would occur through an increase of vasoconstriction or decrease of endothelial vasodilation. Q7 significantly increased the vascular contractile response to phenylephrine in the intact aortic rings and increased intracellular calcium levels in vascular smooth muscle cells (A7r5). Interestingly, the preincubation with ascorbate before addition of Q7 blunted the increase of intracellular calcium caused by Q7 and significantly improved the endothelial vasodilation in the intact aortic rings of the rat. We observed that ACh-induced endothelial vasodilation was significantly decreased by Q7 but the vasodilation was reestablished with the addition of SNP (a NO donor) into intact rat aorta preparations in previously published data [19]. In the same previous study, we demonstrated that Q7 significantly reduced the ACh-induced NO generation in the intact aortic rings [19]. Other studies had reported that the preincubation of mouse aortic rings with 10^−5^ M juglone (5-hydroxy-1,4-naphthoquinone) decreased NO- and ACh-induced relaxation [30] and incubation of rat aortic rings with ascorbate restored vasodilation in L-nitro-L-arginine hypertensive rats [32].

Oxidative stress caused by Q7 is due to ROS generation through a redox-cycling mechanism [11]. The anion superoxide (O_2_^−^) induced by Q7 would react with endothelial NO and could produce peroxynitrite (ONOO^−^). Quinones (i.e., doxorubicin and menadione) increase the generation of ROS, leading to the scavenging of NO [33–35]. Therefore, the decrease in the bioavailability of endothelial NO in blood vessels can explain the increase of the blood pressure as described above [19].

Ascorbate acts as a powerful antioxidant decreasing the oxidative stress in blood vessels by blunting the generation of ROS. Reduced levels of hydroxyl radical (HO^−^) and O_2_^−^ would result in a higher bioavailability of endothelial NO [17]. This is in agreement with reports that ascorbate reduces the level of ROS and improves the endothelial vasodilation in chronic smokers [36].

We found that oral treatment of rats with ascorbate attenuated the elevation of ST segment in ECG and irregular beating of the atrium induced by Q7. The elevation of ST segment represents a partial depolarization in the damaged cells of the heart, compared with the healthy myocardium [37]. Others studies reported that melatonin decreases the elevation of the ST segment on adriamycin-induced cardiotoxicity in rats [38]. To investigate if the cardiac activity in the presence of Q7 could be modulated by changes in intracellular calcium, further experiments were conducted in neonatal rat cardiac myocytes. The results showed that Q7 increased the intracellular calcium levels in rat cardiomyocytes and this Q7-mediated effect was not prevented by preincubation with ascorbate. Conversely, studies have shown that the preincubation with salvianolic acid B for 6 h prevented the doxorubicin-induced increase of the intracellular calcium levels and contractility in cardiomyocytes of rats [39]. Our results do not show a similar effect as that reported for salvianolic acid B, although it could be due to the lower preincubation time used in our experiments (few seconds prior to Q7 addition).

Since 1,4-naphthoquinone induces ROS by a redox-cycling mechanism [11] and Q7 is a 1,4-naphthoquinone derivative, the levels of TBARS, SOD-like activity, and H_2_O_2_ were determined in our experimental models. Although Q7 significantly increased oxidative stress (increased TBARS and decreased SOD-like activity) in serum samples of treated rats, it did not increase the production of H_2_O_2_ in vascular smooth muscle cells of the rat aorta (A7r5). Therefore, our data suggest that Q7 generated O_2_^−^ and increased TBARS, but not H_2_O_2_. One explanation is that the formation of O_2_^−^ and H_2_O_2_ induced by Q7 does not occur simultaneously. Alternatively, the low activity levels of superoxide dismutase enzyme (SOD) in cultured cells could mask the generation of H_2_O_2_ by Q7 [40], although this is unlikely as Q7 decreased SOD-like activity in serum samples.

Our results showed that high concentrations of ascorbate (0.125, 0.25, and 2 mM) significantly increased the generation of H_2_O_2_ in a dose-dependent manner in A7r5 cells. The formulation of culture medium (Dulbecco's modified Eagle's medium (DMEM)) has iron (0.25 mM Fe (NO_3_)_3_), which may act as an essential catalyzer through the process of autoxidation of ascorbate, leading to the higher generation of H_2_O_2_ [41].

Therefore, the route of administration of ascorbate is very important because of the potential generation of ROS. Intravenous infusion of high doses of ascorbate will increase the generation of ROS in the presence of serum iron; as such, ascorbate is considered a prooxidant substance [42] and useful for cancer treatment. Intraperitoneal or intravenous administration of ascorbate could peak concentrations between 3 and 8 mM in blood samples from rats [43]. In contrast, oral administration of high doses of ascorbate would achieve peaks up to 150 M in blood samples from rats [44].

We found that oral administration of ascorbate prevented the increase of Q7-induced TBARS levels and the decrease of SOD-like activity when compared to the Q7-treated group. The 4-hydroxyaniline substituent in Q7 has a low electron donating capacity and is similar to 1,4-naphthoquinone [45], leading to a lower O_2_^−^ formation. In contrast, menadione (2-methyl-1,4-naphthoquinone) has a high electron donating capacity, leading to a higher O_2_^−^ formation [40]. Our findings of TBARS levels and SOD-like activity could be attributed to the partial stabilization of the semiquinone radical species of Q7 by ascorbate, causing a change of redox-cycling process and a lower ROS generation.

In conclusion, oral treatment with ascorbate reduced the Q7-induced increase in blood pressure. This finding is consistent with a putative model in which ascorbate decreased the heart rate and improved endothelial vasodilation in quinone-treated rats. These are possibly mediated by an increase of the endothelial NO bioavailability and reduced calcium-dependent vascular contractile response in vascular smooth muscle cells. These results suggest an *in vivo* cardioprotective effect of oral ascorbate in animals treated with naphthoquinone derivative, which is dependent on oxidative stress. *In vivo*, ascorbate partially reduced the Q7-induced oxidative stress. These findings could have interesting and potential clinical effects for a number of pathologies such as inflammatory disorders, diabetic blindness, cardiovascular and autoimmune diseases, and cancer [46]. More studies with naphthoquinones on cancer models may provide a better understanding of the mechanism underlying the amelioration of cardioprotection by ascorbate.

## Figures and Tables

**Table 1:** Ascorbate decreases intracellular calcium levels in the presence of Q7 in vascular smooth muscle cells (A7r5). The A7r5 cells were preincubated with 10^−5^ M Q7 and/or 2 mM ascorbate (Asc) for 20 min.

	Control	Q7	Q7+Asc	Asc
Ca^2+^ signal (Fluo-3 fluorescence)	209 ± 15	296 ± 23^∗^	230 ± 16	201 ± 10

Values are mean ± standard error of the mean of 3 experiments. Statistically significant differences: ^∗^*p* < 0.05 versus control.

**Table 2:** Oral treatment with Q7 causes oxidative stress in rats. The SOD-like activity represents the autoxidation rate of pyrogallol in an experimental sample and control sample. When the activity is ≥100%, pyrogallol autoxidation is completely inhibited, while the negative value means that it is accelerated by adding Q7.

	Control	Q7	Q7 + Asc	Asc
TBARS (nM)	26 ± 1	33 ± 2^∗^	30 ± 3	25 ± 1
SOD-like activity (%)	159 ± 49	−193 ± 100^∗^	−36 ± 10	150 ± 39

Values are mean ± standard error of the mean of 5 experiments. Statistically significant differences: ^∗^*p* < 0.05 versus control.

## Data Availability

The data used to support the findings of this study are available from the corresponding author upon request.
